# Gastrointestinal adverse effects of varenicline at maintenance dose: a meta-analysis

**DOI:** 10.1186/1472-6904-11-15

**Published:** 2011-09-28

**Authors:** Lawrence K Leung, Francis M Patafio, Walter W Rosser

**Affiliations:** 1 Centre of Studies in Primary Care, Queen's University, 220 Bagot Street, Kingston Ontario, K7L 5E9, Canada; 2 Department of Family Medicine, Queen's University, 220 Bagot Street, Kingston Ontario, K7L 5E9, Canada; 3 School of Medicine, Queen's University, Kingston General Hospital, 18 Stuart Street, Kingston Ontario, K7L 3N6, Canada

## Abstract

**Background:**

Tobacco smoking remains the leading modifiable health hazard and varenicline is amongst the most popular pharmacological options for smoking cessation. The purpose of this study is to critically evaluate the extent of gastrointestinal adverse effects of varenicline when used at maintenance dose (1 mg twice a day) for smoking cessation.

**Methods:**

We conducted a meta-analysis of randomised controlled trials published in PUBMED and EMBASE according to the PRISMA guidelines. Selected studies satisfied the following criteria: (i) duration of at least 6 weeks, (ii) titrated dose of varenicline for 7 days then a maintenance dose of 1 mg twice-per-day, (iii) randomized placebo-controlled design, (iv) extractable data on adverse event - nausea, constipation or flatulence. Data was synthesized into pooled odd ratios (OR) basing on random effects model. Quality of studies was also rated as per Cochrane risk-of-bias assessment. Number need to harm (NNH) was calculated for each adverse effect.

**Results:**

98 potentially relevant studies were identified, 12 of which met the final inclusion criteria (n = 5114). All 12 studies reported adverse events on nausea, which led to an OR of 4.45 (95% CI = 3.79-5.23, *p *< 0.001; I^2 ^= 0.06%, CI = 0%-58.34%) and a NNH of 5. Eight studies (n = 3539) contain data on constipation pooled into an OR of 2.45 (95% CI = 1.61-3.72, *p *< 0.001; I^2 ^= 34.09%, CI = 0%-70.81%) with a NNH of 24. Finally, five studies (n = 2516) reported adverse events of flatulence, which pooled an OR of 1.74 (95% CI = 1.23-2.48, *p *= 0.002; I^2 ^= 0%, CI = 0%- 79.2%) with a NNH of 35.

**Conclusions:**

Use of varenicline at maintenance dose of 1 mg twice a day for longer than 6 weeks is associated with adverse gastrointestinal effects. In realistic terms, for every 5 treated subjects, there will be an event of nausea, and for every 24 and 35 treated subjects, we will expect an event of constipation and flatulence respectively. Family physicians should counsel patients of such risks accordingly during their maintenance therapy with varenicline.

## Background

Tobacco smoking remains the most modifiable risk factor for premature death and all-cause mortality [1], with a global estimate of 4.83 million attributable deaths in year 2000 [2]. As a result, many pharmacological agents have been developed to help patients stop tobacco smoking, some of which can be administered either nasally, orally (chewable gum or tablets) or topically (e.g., nicotine replacement patch). Amongst these agents, varenicline (branded as Champix^© ^in UK and Canada, Chantix^© ^in USA) has been shown to be the one of the most effective oral pharmacological agents for continued abstinence. Varenicline acts as a partial α_4_β_2 _nicotinic acetylcholine receptor agonist in the brain to potentially decrease the degree of cravings and withdrawal symptoms during the period of smoking cessation, with effects superior to both placebos and other pharmacological agents [3]. Animal studies have found that tobacco addiction is mediated via nicotinic acetylcholine receptors (nAChRs) in the meso-limbic area of the brain that contain α_4 _and β_2 _subunits. While α_4 _subunits are needed for sensitization and reinforcement to nicotinic effects and their tolerance, involvement of β_2 _subunits are indispensable for development of dependence [4]. In vivo studies have shown that in the presence of nicotine, varenicline acts as a partial agonist which stimulates the release of dopamine (to ameliorate the symptoms of craving and withdrawal) and simultaneously block the nicotine receptors (to reduce the likelihood of dependence)[5,6]. When compared to other mainstream pharmacotherapies (e.g., nicotine replacement therapy and bupropion), varenicline remains as the most effective choice for smoking cessation [7] and is validated by high-quality meta-analysis [8]. Nevertheless, varenicline has been associated with adverse events like headache, fatigue, sleep disorder, nausea and constipation [9,10]. Whilst risks of neuropsychiatric adverse events due to varenicline have already been reported in a polled analysis [11], the likelihood of major gastrointestinal adverse effects (such as nausea, constipation and flatulence) during maintenance phase lacks precise documentation. We hereby performed a meta-analysis of randomized double-blinded placebo-controlled trials to critically examine the relative risks of nausea, constipation and flatulence due to maintenance dose of varenicline (1 mg twice a day for at least 6 weeks) in the context of smoking cessation.

## Methods

### Eligibility criteria

Our primary interest was the reported gastrointestinal adverse-effects in using varenicline at the indicated maintenance dose (1 mg) for duration longer than six weeks. To minimise heterogeneity, we limited our scope to double-blind randomised placebo-controlled trials with comparable sample sizes and a satisfactory score from the Cochrane risk of bias assessment tool described by Higgins [12]. Eligible studies must use a one-week titrated dose (0.5 mg) of varenicline prior to maintenance dose (1 mg twice a day), and contain at least one extractable adverse gastrointestinal event of nausea, constipation or flatulence.

### Search strategy

A literature search of published medical reports was performed in all languages from PUBMED (from 1947 to December 2010), EMBASE (from 1947 to December 2010) and All EBM Reviews using the OVID Portal of Queen's University, Kingston, Ontario. Abstracts were initially obtained using keywords of "smoking cessation" AND "varenicline. They were further narrowed down by imposing keywords of "human" AND "controlled trial". Manual searches of references and review articles supplemented the computerized search.

### Study selection, data extraction and quality assessment

Two reviewers (LL and FP) worked independently and went over the initial search for abstracts that satisfied the keywords as mentioned in the search strategy section. They then adopted a simple form to select trials that satisfied the eligibility criteria stated above. Evaluation of selected studies were performed independently by each reviewer according to the Cochrane risks of bias tool [12] in regards to the quality of study, randomization protocol, adequacy of concealment and blinding and, rigor of follow-up for dropouts. Information was extracted and tabulated in spreadsheet regarding the demography of the study population, duration of study, the types of adverse events, the number of affected subjects taking varenicline and placebo respectively and finally a numeric score for the Cochrane risk of bias. Spreadsheets were compared and any disagreement was discussed and resolved to reach mutual consensus.

### Statistical Analysis

All data were synthesized in a meta-analysis and odds ratios (OR) were calculated with appropriate confidence intervals (CI) basing on the number of subjects reporting the relevant adverse effects in the study. Where necessary, the value of 1 was added to any arm with zero outcome event according to the Sheele +1 rule [13]. The random effects analysis model as described by DerSimonian and Laird [14] was adopted instead of the fixed effects model to account for extra variance due to heterogeneous samples drawn from a wide population [15]. Forest plots [16] were generated basing on OR for each gastrointestinal symptom of nausea, constipation and flatulence. Basing on the values of OR and the baseline risks of adverse effects, the number needed to harm (NNH) was also calculated which gives a realistic idea of the likelihood of the adverse effects. Funnel plots [17] were displayed as a reference for possible publication bias. To assess heterogeneity across included studies, we adopted the Cochran Q-statistic [18] and the I^2 ^index [19,20] with 95% confidence intervals. We assumed a *p*-value of less than 0.10 for the Cochran Q-statistic and an I^2 ^index of greater than 50% as a threshold of heterogeneity. Statistical advice was provided by data analyst at our Centre of Studies in Primary Care.

## Results

### Study description

**Figure 1:**
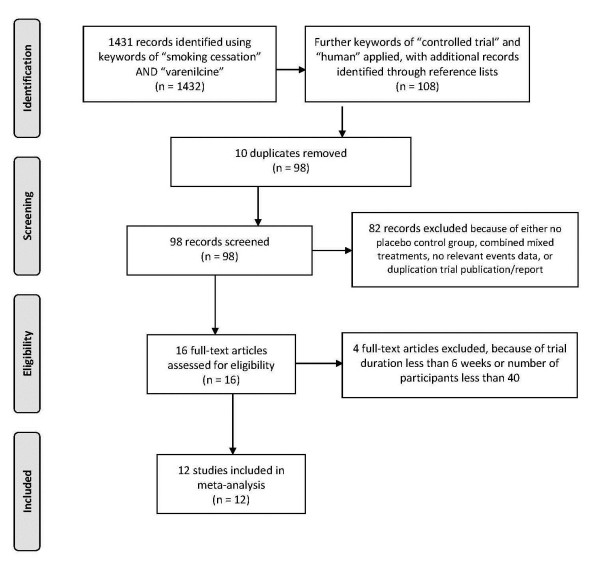
**Flow diagram (in PRISMA format) of literature search**.

1431 abstracts were identified using keywords of "smoking cessation" AND "varenicline", which were reduced to 108 abstracts when imposing extra keywords of "human" AND "controlled trial" with supplementation from reference lists of included abstracts. 10 duplicates were removed and 82 abstracts were further excluded on grounds of having no placebo control group, combined mixed treatments, no relevant gastrointestinal adverse events data, or duplication trial publication or report. 16 relevant full text paper publications were then retrieved for potential inclusion. After imposing the eligibility criteria as described, only 12 studies were deemed eligible upon which mutual consensus was reached between the reviewers after discussion and comparison of the data spreadsheets. Using the 2009 PRISMA checklist [21] (Additional File 1), all 27 items were present and identified scored. A flow diagram of the literature search and selection process according to the PRISMA format [21] was given in Figure 1, and the included studies were tabulated in Table 1. From the 12 studies, 2,622 patients were randomly assigned to receive varenicline (oral dose of 1 mg twice per day), and 2,492 patients were randomly assigned to receive placebo. Total sample size per study ranged from 248 to 714 and three studies had a total size of greater than 500. Except for the study by Tashkin et al. [22] which specifically targeted patients with COPD, all other studies recruited subjects from the general population. One trial used varenicline for 6 weeks [23] while another for 52 weeks [24], the rest used 12 weeks. All but one study [23] employed the standard low-dose titration regime before using the 1 mg twice per day dosage. There was no exclusion as for the type of tobacco used by smokers in all trials except for the study by Fagerstrom et al. [9] which recruited subjects using smokeless tobacco.

**Table 1:** Summary of the 12 studies included for meta-analysis

Study	Total Sample Size(n)	**Adverse effects**^**¥**^	Mean Age	% Male	Trial Duration with 1 mg BID dose(Weeks)	Size of Trial group	Size of Control Group	**Cochrane risk of bias assessment**^**§**^						
								
								P	Q	R	S	T	U	Overall score
**Gonzales et al (2006)**^**32 **^	696	A, B, C	42.55	52.05	12	352	344	1	1	1	1	1	1	6/6
**Jorenby et al(2006)**^**29 **^	685	A, B, C	43.45	56.65	12	344	341	1	1	1	1	1	0	5/6
**Oncken et al(2006)**^**27**^	259	A, B, C	41.1	50.2	12	130	129	1	1	1	1	0	0	4/6
**Nides et al(2006)**^**25**^	248	A, B	41.7	51.2	6	125	123	1	1	0	1	1	0	4/6
**Nakamura et al(2007)**^**30**^	310	A, B	40	77.6	12	156	154	1	1	1	1	1	1	6/6
**Tsai et al(2007)**^**28**^	250	A, B	40.3	88.8	12	126	124	1	1	0	1	1	0	4/6
**Williams et al(2007)**^**26**^	377	A, B, C	47.7	48.6	52	251	126	1	1	0	1	1	0	4/6
**Niaura et al(2008)**^**34**^	312	A	41.8	51.9	12	157	155	1	1	1	1	1	0	5/6
**Wang et al(2009)**^**33 **^	333	A	38.7	96.7	12	165	168	1	1	0	1	1	0	4/6
**Rigotti et al(2010)**^**31**^	714	A, B	56.45	78.7	12	355	359	1	1	0	1	1	1	5/6
**Fagerstrom et al(2010)**^**8**^	431	A	43.9	89.3	12	213	218	1	1	1	1	1	1	6/6
**Tashkin et al(2010)**^**24 **^	499	A, C	57.2	62.3	12	248	251	1	1	0	1	1	0	4/6

^¥^A = Nausea, B = Constipation, C-Flatulence

^#^Denote use of 1-week low-dose titration treatment

^§^Cochrane risk of bias assessment: P = Allocation sequence adequately generated?; Q = Allocation adequately concealed?; R = Knowledge of allocated intervention adequately concealed?; S = incomplete outcome data adequately addressed?; T = Reports free of selective outcome reporting?; U = study free of other factors leading to high risk of bias?; 1 = item positive; 0 = negative or unknown.

### Assessment of study quality

All 12 studies achieved a score of at least 4 out of 6, using the Cochrane risk of bias assessment [12] (See table 1) and was considered satisfactory with mutual agreement between the two reviewers. After consultation with our statistician, the random effects model was adopted for subsequent analysis.

### Outcome Measures

#### Nausea

**Figure 2:**
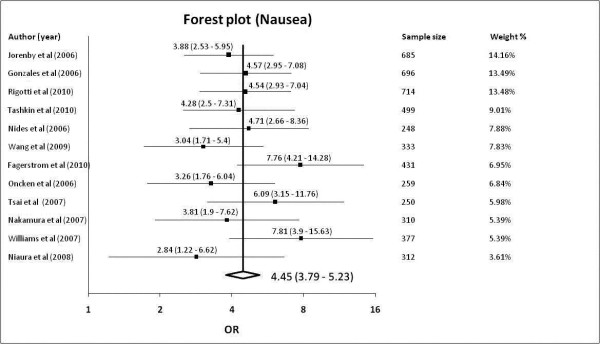
**Forest plot showing odds ratio for nausea**.

All 12 studies [9,22-32] reported adverse effects of nausea with a total sample size of 5114. Out of the total 2622 subjects randomized to varenicline, 826 reported nausea; of the 2492 subjects taking placebo, 232 had nausea. The pooled OR was 4.45 (95% confidence levels [CI] of 3.79-5.23, *p *< 0.001) with a non-significant Cochrane Q-statistic of 11.01 (*p *= 0.443) and an I^2 ^index of 0.06% (95% confidence levels of 0% - 58.34%). With a baseline risk of nausea at 9.3%, the number need to harm (NNH) is 5. The Forest plot was shown in Figure 2 and funnel plot in Additional File 2.

#### Constipation

**Figure 3:**
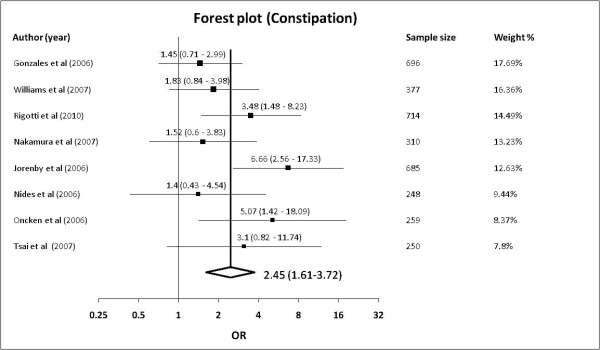
**Forest plot showing odds ratio for constipation**.

8 studies [23-30] reported adverse effects of constipation with a total sample size of 3539. 146 of 1839 subjects in the varenicline group and 53 of 1700 in the control group reported adverse effects of constipation. The pooled OR was 2.45 (95% confidence levels [CI] of 1.61-3.72, *p *< 0.001) with a non-significant Cochrane Q-statistic of 10.62 (*p *= 0.156) and an I^2 ^index of 34.09% (95% confidence levels of 0% - 70.81%). The baseline risk of constipation is 3.1%, yielding a NNH of 24. The Forest plot was shown in Figure 3 and the funnel plot in Additional File 2.

#### Flatulence

**Figure 4:**
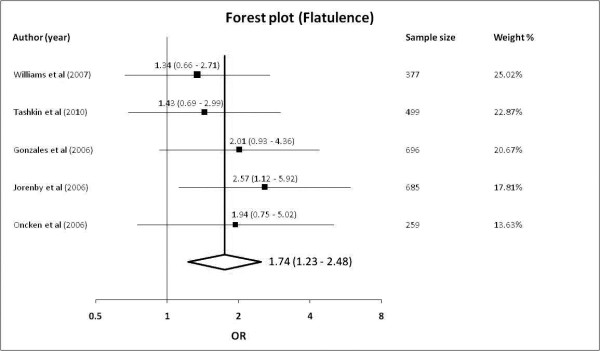
**Forest plot showing odds ratio for flatulence**.

5 studies [22,24,25,27,30] were included reporting adverse effects of flatulence with a total sample size of 2516 subjects. 102 of 1325 subjects in the varenicline group and 50 of 1191 in the control group reported adverse effects of flatulence. The pooled OR was 1.74 (95% confidence levels [CI] of 1.23-2.48, *p *= 0.002) with a non-significant Cochrane Q-statistic of 1.82 (*p *= 0.768) and an I^2 ^index of 0% (95% confidence levels of 0% - 79.2%). With a baseline risk of flatulence at 4.2%, the NNH is 35. The Forest plot was shown in Figure 4 and the funnel plot in Additional File 2.

## Discussion

Our meta-analysis data confirms that using varenicline at maintenance dose of 1 mg twice per day for a period of more than 6 weeks is significantly associated with adverse effects of nausea and, to a lesser extent, constipation and flatulence. Using pooled odds ratios (OR) and subsequently translating them to number need to harm (NNH), we showed that for nausea, constipation and flatulence, the NNH is 5, 24 and 35 respectively. Such numbers provide clinicians with a realistic picture of the likelihood for gastrointestinal adverse effects when prescribing varenicline as a drug of choice for smoking cessation. As the commonest gastrointestinal adverse effect, nausea accounts for failure of varenicline treatment amongst 1.8% to 7.6% of our study population [24,26,27,30,31]. The effects of nausea usually peak around 4 weeks of treatment and becoming less thereafter [24]. No similar discontinuation rates due to effects of constipation or flatulence have been reported.

### Limitation of studies

Our study has two limitations. Gastrointestinal adverse effects like nausea and flatulence are primarily subjective feelings which are difficult to quantify, despite crude stratification into mild, moderate or severe in several studies. Definition for constipation is also not standardised as per Rome criteria [33] amongst the included studies in terms of bowel frequency or patient's difficulty in defecation. They may constitute data heterogeneity across studies in our meta-analysis.

The second limitation is the potential of publication bias. Our search strategy only isolated full-length published trials with sufficient population sizes to maintain statistical significance. In addition, we excluded studies with populations less than 40. However adoption of the random effects model in meta-analysis will help compensate irregularities due to small and potentially negative studies that we have excluded. Our funnel plots do not show obvious asymmetry (Additional File 2), suggesting the low likelihood of publication bias in our analysis. Thus said, the visual asymmetry of funnel plots should not be used to confirm the extent of publication bias [34], especially when the number of studies is small [35]. The I^2 ^index suggests very low heterogeneity across studies for both nausea and flatulence (both at 0%) and slightly higher but still acceptable heterogeneity for constipation (34%)[20]. Like the Cochran Q-statistic, the I^2 ^index is known to have limitations with its power affected by the actual number of included studies [36].

## Conclusions

Varenicline is one of the most preferred pharmacological options for smoking cessation and gastrointestinal adverse effects have been mentioned but not documented precisely. Our comprehensive meta-analysis on randomised double-blind placebo-controlled trials concluded that in realistic terms, use of varenicline at the indicated maintenance dose (1 mg twice per day) for longer than 6 weeks will lead to one adverse event of nausea for every 5 treated subjects, one event of constipation for every treated 24 subjects and one event of flatulence for every 35 treated subjects. These data will better enable clinicians in counseling patients when using varenicline for smoking cessation both in facilitating and reinforcing optimal smoking cessation rate.

## Conflict of interests

The authors declare that they have no competing interests.

## Authors' contributions

LL and FP designed the review and performed the literature search. LL performed the analysis and generation of the graphic plots. LL and FP analysed and interpreted the data and both contributed to the drafting of the manuscript. WR provided comments and advice on the overall study. ALL authors have read and approved the submitted version for publication.

## Pre-publication history

The pre-publication history for this paper can be accessed here:

http://www.biomedcentral.com/1472-6904/11/15/prepub

## Supplementary Material

Additional file 1 **PRISMA 2009 Checklist for the meta-analysis study**. All items in the 2009 PRISMA checklist are considered and verified against the page of the original manuscript. Click here for file

Additional file 2 **Funnel plots showing bias of studies for adverse effects of (a)nausea, (b)constipation, (c)flatulence**. File contains the funnel plots of various studies basing on the odds ratio, as categorised by the adverse effects of nausea, constipation and flatulence respectively. Click here for file
