# Supplementary figures and images for: Gastrointestinal adverse effects of varenicline at maintenance dose: a meta-analysis
Source: BMC Clin Pharmacol. 2011 Sep 28;11:15. doi: 10.1186/1472-6904-11-15 (PMC3192741; doi:10.1186/1472-6904-11-15)

(a)
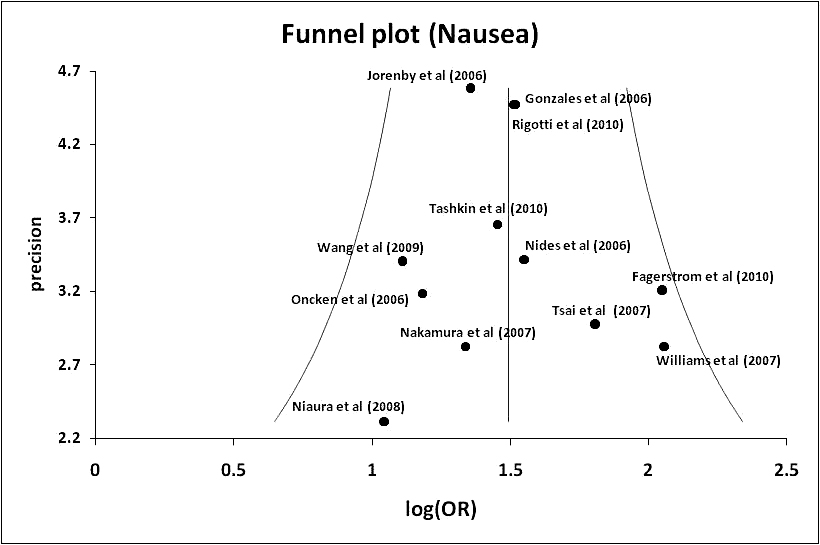


(b)
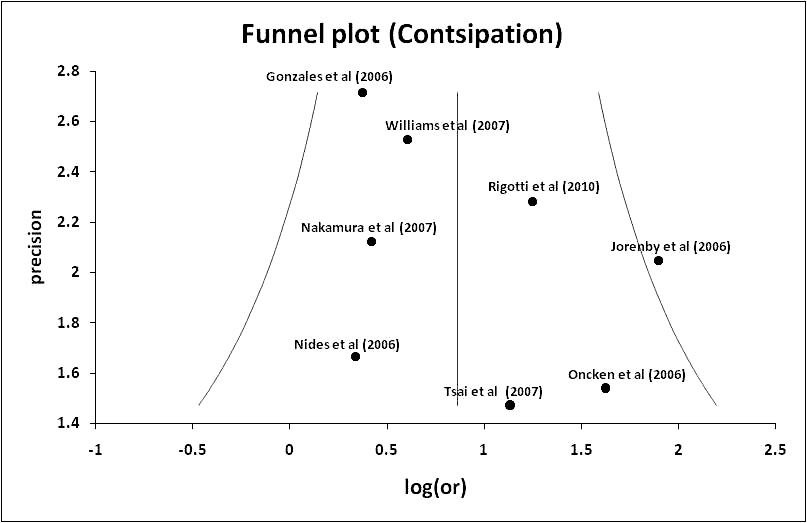


(c)
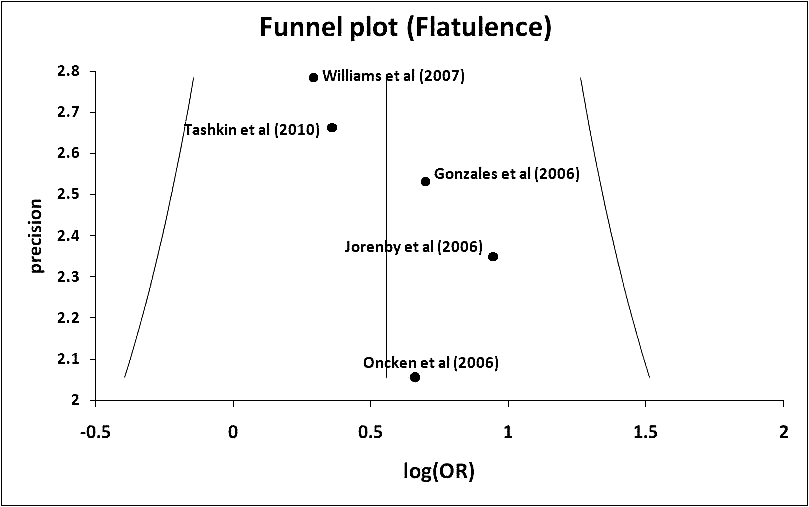

Supplement: Additional file 2 — Funnel plots showing bias of studies for adverse effects of (a)nausea, (b)constipation, (c)flatulence. File contains the funnel plots of various studies basing on the odds ratio, as categorised by the adverse effects of nausea, constipation and flatulence respectively. [file 1472-6904-11-15-S2.DOC]
